# Peripheral blood mononuclear cells extracts VEGF protein levels and VEGF mRNA: Associations with inflammatory molecules in a healthy population

**DOI:** 10.1371/journal.pone.0220902

**Published:** 2019-08-16

**Authors:** Vesna Gorenjak, Dwaine R. Vance, Alexandros M. Petrelis, Maria G. Stathopoulou, Sébastien Dadé, Said El Shamieh, Helena Murray, Christine Masson, John Lamont, Peter Fitzgerald, Sophie Visvikis-Siest

**Affiliations:** 1 Université de Lorraine, Inserm, IGE-PCV, Nancy, France; 2 Randox Laboratories Limited, Crumlin, Co. Antrim, Northern Ireland, United Kingdom; 3 Department of Medical Laboratory Technology, Faculty of Health Sciences, Beirut Arab University, Beirut, Lebanon; 4 Department of Internal Medicine and Geriatrics, CHU Technopôle Nancy-Brabois, Rue du Morvan, Vandoeuvre-lès-Nancy, France; Medical University Innsbruck, AUSTRIA

## Abstract

**Background:**

Vascular endothelial growth factor (VEGF) is a signal protein, implicated in various physiological and pathophysiological processes together with other common inflammatory biomarkers. However, their associations have not yet been fully elucidated. In the present study, we investigated associations between VEGF and four specific *VEGF* mRNA isoforms with levels of 11 inflammation molecules, derived from peripheral blood mononuclear cells (PBMCs) extracts.

**Methods:**

Healthy participants from the STANISLAS Family Study (n = 285) were included. Levels of VEGF (four mRNA isoforms and protein levels) and inflammatory molecules (IL-1α, IL-1β, IL-2, IL-4, IL-6, IL-8, IL-10, INF-γ, TNF-α, MCP-1, EGF) were measured in PBMCs extracts. Multiple regression analyses were performed, adjusted for age and gender.

**Results:**

The analyses revealed significant associations between VEGF protein levels and levels of IL-4 (β = 0.028, P = 0.013), MCP-1 (β = 0.015, P<0.0001) and EGF (β = 0.017, P<0.0001). Furthermore, mRNA isoform *VEGF*_*165*_ was associated with MCP-1 and IL-1α (P = 0.002 and P = 0.008, respectively); and mRNA isoform *VEGF*_*189*_ was associated with IL-4 and IL-6 (P = 0.019 and P = 0.034, respectively).

**Conclusions:**

To our knowledge, the present study represents the first investigation that successfully demonstrates links between VEGF protein levels and inflammatory molecules levels derived from PBMCs extracts and identifies associations between specific *VEGF* mRNA isoforms and inflammatory molecules.

**Impact:**

These findings provide novel insights that may assist in the development of new tissue and mRNA isoform specific measurements of VEGF levels, which may positively contribute to predicting the risk of common complex diseases and response of currently used anti-VEGF agents, and developing of novel targeted therapies for VEGF-related pathophysiology.

## Introduction

Vascular endothelial growth factor A, VEGF-A (commonly referred as VEGF), is a multifunctional signal protein, which works as an important regulator of both physiological and pathological angiogenesis and has been related to a variety of pathologies, such as cancer and cardiovascular diseases (CVD) [1]. VEGF has become a perspective target for the design of anti-cancer treatments; anti-VEGF medications have already entered the clinical environment, however, the trade-off for the therapy is a common occurrence of cardiovascular side effects [2, 3].

VEGF is a prototype member of a cytokine family, which also includes placental growth factor (*PLGF*), VEGF-B, VEGF-C and VEGF-D, present in regulation of lymphangiogenesis, vasodilatation, chemotactic for different cells and vascular permeability [1]. Levels of VEGF represent a highly heritable phenotype (>60.5% as demonstrated in STANISLAS Family Study (SFS)) [4]. More than 50% of this variability is explained by ten single nucleotide polymorphisms identified through two genome-wide association studies (GWAS) [5, 6].

The *VEGF* gene produces more than 14 messenger RNA (mRNA) isoforms; the most predominant are *VEGF*_*121*_, *VEGF*_*145*_, *VEGF*_*165*_ and *VEGF*_*189*_, denoted by their length (number of amino acids). These isoforms differ in biochemical properties and receptor-binding characteristics that result in different effects on vessel growth [7]. Various tissues express different ratios of *VEGF* mRNA isoforms, including tumours, where growth appears to be most rapid when the isoform *VEGF*_*164*_ is expressed [7]. Therefore, disease susceptibility may depend on transcription of specific *VEGF* mRNA isoforms, rather than the currently measured VEGF protein. As a result, studies exploring specific *VEGF* mRNA isoforms for association with inflammatory molecules, intermediate phenotypes and diseases are warranted.

Human VEGF isoforms are classified into two main families: the *VEGFxxx* family and the *VEGFxxxb* family (xxx denoting the number of amino acids) [8]. The isoforms of these families differ only in the sequence of carboxy-terminal six amino acids, as the result of alternative splicing of exon eight of the *VEGF* gene [9]. This difference leads to isoforms with opposite functions; VEGFxxx isoforms (e.g. VEGF_165_) have pro-angiogenic properties whereas the VEGFxxxb isoforms (e.g. VEGF_165b_) have anti-angiogenic properties [10, 11]. VEGFxxxb isoforms present more than half of the total VEGF expressed in vitreous fluid, circulating plasma, urine, renal cortex, colonic epithelium, bladder smooth muscle, lung and pancreatic islets, whereas in tissues with physiological angiogenesis (placenta) or pathological angiogenesis (melanoma, colorectal or bladder cancer cells) VEGFxxx isoforms represent the majority of total VEGF [8].

VEGF is secreted from various cells: fibroblasts, tumour cells and inflammatory cells, such as lymphocytes [12, 13]. Lymphocytes are the most numerous components of peripheral blood cells (70–90% of PBMCs) and hold a central role in the regulation of the immune system [14]. Originating from a common lymphoid progenitor, there are three subgroups of lymphocytes, namely T-lymphocytes (70–85%), B-lymphocytes (5–20%) and natural killer cells (NK) (5–20%), each with their own function in immune response [15, 16]. Chronic inflammation often results in the infiltration of inflammatory cells at specific sites, among which the predominant are T-lymphocytes [17]. In addition to VEGF, other important signalling proteins are secreted from lymphocytes and are regulated by intracellular signalling control mechanisms or extracellular regulators [18]. Together, they are involved in complex molecular pathways that impact on physiological balance in human organism. The most important cytokine families involve hematopoietins, interferons, tumour necrosis factors-related molecules and chemokines [19]. VEGF levels have been previously related with cellular adhesion molecules (CAMs) [20], interleukin-1α (IL-1α)[21], interleukin-1β (IL-1β) [22], interleukin-4 (IL-4) [23–25], interleukin-6 (IL-6) [26, 27], nuclear factor-κB (NF-κB) transcription factor [28], endothelial growth factor (EGF) and others [29–31].

A detailed knowledge of biological pathways of VEGF is indispensable for further progress in pharmacological studies and detection of different isoforms of VEGF is of crucial importance for anticancer therapy with limited side effects. In addition, unravelling the relations between different biomarkers in physiological state of organisms can provide the basic knowledge that will help to understand these relations in pathological conditions.

The aim of this investigation is to assess the relationship between VEGF and inflammatory molecules, including cytokines and growth factors: IL-1α, IL-1β, IL-2, IL-4, IL-6, IL-8, IL-10, interferon-γ (INF-γ), tumour necrosis factor α (TNF-α), monocyte chemotactic protein 1 (MCP-1) and EGF, derived from PBMCs extracts in healthy individuals. PBMCs represent one of the most important sources of inflammatory molecules; therefore, we examined associations of cytokines and growth factors contained within PBMCs extracts utilizing a multiplex chemiluminescent biochip. Furthermore, because of the importance of VEGF isoforms, we examined the mRNA expression profiles of the four most biologically important VEGF isoforms (*VEGF*_*121*_, *VEGF*_*145*_, *VEGF*_*165*_ and *VEGF*_*189*_) and explored associations of each specific *VEGF* mRNA isoform with inflammatory molecules.

## Materials and methods

### Population

All subjects involved in this study make part of the STANISLAS Family Study (SFS). Information pertaining to this cohort has previously been described [32, 33]. Briefly, the SFS is a 10-year longitudinal survey with 3 visits at 5-year intervals, involving 1,006 families from Vandoeuvre-lès-Nancy, France, first recruited between 1993 and 1995. All subjects were of Northwest-European origin, without the presence of chronic disorders *e*.*g*. CVD or cancer, and without previous personal history of CVD. Study protocols were approved by the institutional ethics committees CCPPRB de Lorraine (Comité consultatif de protection des personnes dans la recherche biomédicale) and CNIL (Commission Nationale de l'Informatique et des Libertés). All individuals gave written informed consent for their participation in the study.

A subset of 285 participants (138 females and 147 males) from the SFS was selected for the measurement of twelve inflammatory molecules derived from PBMCs extracts.

A subset of 110 participants from the SFS was selected to quantify four specific *VEGF* mRNA isoforms from total RNA derived from PBMCs (*i*.*e*. *VEGF*_*121*_, *VEGF*_*145*_, *VEGF*_*165*_ and *VEGF*_*189*_), based on the sample availability.

### Laboratory measurements

#### Isolation of PBMCs

Isolation of PBMCs was based on the method first described by Boyum in 1968 [34]. Briefly, whole blood was collected in tubes with sodium heparin and transported at room temperature. Hanks’ Balanced Salt Solution (SIGMA Aldrich, reference: H6648) was added into 15 mL tubes with blood (V_Hanks_ = V_blood_) and poured gently into a 15 mL tube with Ficoll paque plus (Sigma Aldrich, reference: 17-1440-02) solution (V_Ficoll_ = V_Hanks_+ V_blood_). The contents were centrifuged for 30 min at 300 g at room temperature.

A PBMCs ring was retrieved and collected into a 15 mL tube, filled with Hanks’ Balanced Salt Solution and centrifuged for 10 min at 1000 g at room temperature. The supernatant was aspirated and 2 mL of Hanks’ Balanced Salt Solution were added. The solution was well suspended, filled up to 15 mL with Hanks’ Balanced Salt Solution and centrifuged for 10 min at 1000 g at room temperature (second washing). The PBMC ring was collected into Eppendorf tube with 1 mL of Hanks’ Balanced Salt Solution. PBMCs populations were evaluated by microscopic observation after May-Grunwald-Giemsa staining and the PBMCs concentration was normalized to 10^6^ cells/mL in Hank’s Buffer. After final centrifugation 5 min at 1000 g at room temperature the supernatant was aspirated and the pellet of PBMCs was processed immediately or stored at -80 °C to maintain stability.

#### Total protein extraction

The lysis solution (lysate) composed of cell lysis buffer (CelLytic-M, SIGMA Aldrich, reference: C2978) and protease inhibitor (0.5%, Protease Inhibitor Cocktail, SIGMA Aldrich, reference: P8215) was added to the PBMC pellet, as recommended by the manufacturer (SIGMA Aldrich). This was stirred for 15 min at room temperature, and centrifuged for 15 min at 12000 g at 4 °C. The supernatant was collected and was immediately used for further analysis or stored at -80 °C to maintain stability.

#### Protein measurement

Quantification of inflammatory molecules (IL-1α, IL-1β, IL-2, IL-4, IL-6, IL-8, IL-10, MCP-1, TNF-α, INF-γ, EGF and VEGF (combination of VEGF_121_, VEGF_145_, VEGF_165_ and VEGF_189_ isoforms protein levels)) from PBMCs extracts was performed by Randox high sensitivity multiplex cytokine and growth factor array (Evidence Investigator Analyzer, Randox Laboratories Ltd., Crumlin, United Kingdom), which is a Randox patented 9 x 9 mm^2^ activated biochip with spatially discrete test regions containing antibodies specific to each of the inflammatory molecules assessed. In the present study, the combination of the four isoforms measured with the Cytokine array is referred to as “VEGF protein level”.

#### Gene expression analysis

Total RNA was extracted and quantified from isolated PBMCs using the MagNA Pure LC RNA HP isolation kit and RNA HP Blood External lysis protocol (Roche Diagnostics, France) as previously described [35, 36]. In short, 200 units of M-MuLV Reverse Transcriptase with 0.25 μg of oligos (dT) (Promega, France) were used to perform reverse transcription of total RNAs. Quantitative real-time PCR was performed on LightCycler instrument (Roche Diagnostics, Mannheim, Germany) with TaqMan Master Kit for all *VEGF* transcripts. Specific primers were used to selectively quantify the transcripts coding for the *VEGF* isoforms (S1 Table). All quantifications were carried out in duplicates, the starting amount of cDNA template was the same in all samples (25 ng) to remove any bias resulting from difference in the initial RNA quantity. Positive controls with known concentration were included in every run to ensure reproducibility by comparing the expression of *VEGF* transcripts between different PCR runs. A standard curve was generated by plotting the whole range of series dilution against the initial template quantity; this showed a linear standard curve and an efficiency of amplification near 99%. The absolute quantity of *VEGF* transcripts (number of copies/μL) in every sample was calculated by comparing the sample Ct value to the standard curve (Standard Curve Quantification method, The LightCycler 480 Software, Roche Diagnostic, France).

### Statistical analysis

VEGF protein levels were tested for normal distribution using the Kolmogorov-Smirnov test of normality. Consequently, VEGF protein levels were log transformed (log_10_) to normalize a distribution of data. Data was tested for outliers (1.5×IQR interquartile range), which were removed before further analysis. Non-parametric correlation analyses (Spearman correlation) were performed. Linear regression models were applied, adjusted for age and gender, to test for possible associations between VEGF protein levels and inflammatory molecules.

Associations of specific VEGF mRNA isoforms and inflammatory molecules were assessed using identical statistical procedures. Only VEGF isoform 145 values didn’t follow the normal distribution and were log transformed before further analysis.

Mixed models adjusted for family structure were also applied to correct for possible issues of stratification due to familial resemblance.

All analyses were performed using SPSS 20.0 statistical software (SPSS, Armonk, New York: IBM Corp). Significance was determined at a two-tailed 0.05 level. Graphs for correlation and regression analyses were performed using R 3.5.2 statistical software with Jtools package.

### Bioinformatics analysis

Alignment between different isoforms of VEGF was performed using Clustal-Omega program with the following parameters: default transition matrix Gonnet, gap opening penalty 6 bits and gap extension 1 bit. Clustal-Omega uses the HHalign algorithm and its default settings as its core alignment engine. The algorithm is described in details in Söding, J. [37].

## Results

All twelve cytokines were detected in the studied population. All values represent the concentrations of proteins (pg/mL) measured in the cellular extracts. Concentrations are based on equal number of PBMCs (10^6^) in all samples, counted before cell lysis. The characteristics of the study population are presented in Table 1.

**Table 1:** Characteristics of the study population (n = 285).

**Characteristic**	**Mean**	**SD**
**Age (years)**	39.13	14.57
**Gender (%) male**	51.58	-
**Interleukin 1 alpha (pg/mL)**	0.90	4.84
**Interleukin 1 beta (pg/mL)**	5.76	27.61
**Interleukin 2 (pg/mL)**	1.19	1.72
**Interleukin 4 (pg/mL)**	7.59	1.47
**Interleukin 6 (pg/mL)**	0.66	1.43
**Interleukin 8 (pg/mL)**	68.11	147.06
**Interleukin 10 (pg/mL)**	0.64	0.24
**Interferon gamma (pg/mL)**	0.55	3.06
**Tumour Necrosis Factor alpha (pg/mL)**	3.87	16.01
**Monocyte Chemoattractant Protein 1 (pg/mL)**	9.40	5.60
**Epidermal Growth Factor (pg/mL)**	6.80	6.50
**Vascular Endothelial Growth Factor (pg/mL)**	56.99	67.62

SD: Standard deviation

### Associations between VEGF levels and inflammatory molecules

**Fig 1:**
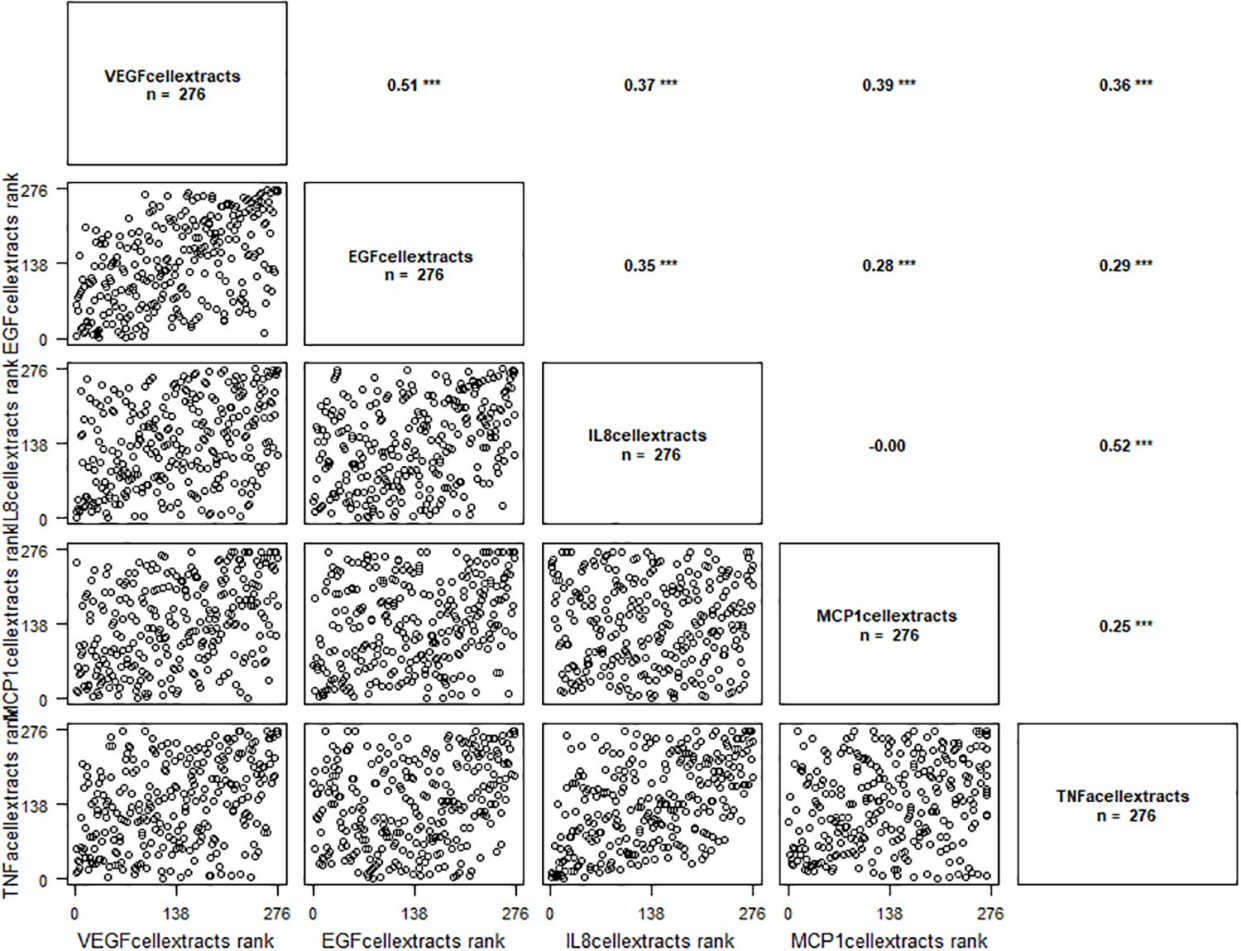
Correlation graph between VEGF protein levels (VEGF cellular extracts) and EGF, IL-8, MCP1 and TNF-α cellular extracts levels. ‘***’ p ≤ 0.001.

VEGF protein levels were correlated with EGF, IL-1β, IL-8, MCP1 and TNF-α (Fig 1). Results are presented in Table 2, the significant P-values are presented in bold.

**Table 2:** Correlation analysis between VEGF protein levels and inflammation molecules.

	VEGF	
	Correlation coefficient	P-value
**EGF**	0.5529	**< .0001**
**IFN-γ**	0.1677	0.2599
**IL-1α**	0.001	0.9945
**IL-1β**	0.2896	**0.0483**
**IL-2**	-0.0911	0.5427
**IL-4**	0.2256	0.1274
**IL-6**	-0.0077	0.959
**IL-8**	0.3393	**0.0197**
**IL-10**	-0.0571	0.7028
**MCP1**	0.3868	**0.0072**
**TNF-α**	0.394	**0.0061**

**Fig 2:**
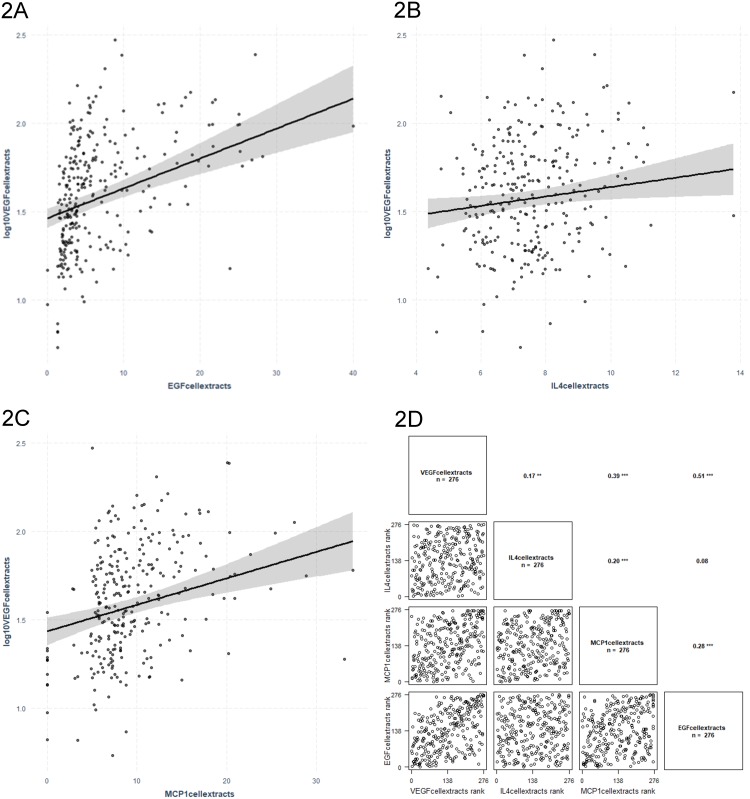
Regression graphs for VEGF protein levels (VEGF cellular extracts) as dependent variable and EGF (2A), IL-4 (2B) and MCP1 (2C) cellular extracts levels as independent variables. The related correlation graph is presented in (2D). ‘***’ p ≤ 0.001, ‘**’ p ≤ 0.01.

VEGF protein levels were associated with IL-4, MCP-1 and EGF levels in linear regression models adjusted for age and gender (Fig 2). Results are presented in Table 3.

**Table 3:** Significant determinants of VEGF protein levels extracted from PBMCs (linear regression analyses adjusted for age and gender).

		VEGF	
Inflammatory molecule	β	*SE*	*P*
**Interleukin 4**	0.028	0.011	0.013
**Monocyte Chemoattractant Protein 1**	0.015	0.003	<0.0001
**Epidermal Growth Factor**	0.017	0.003	<0.0001

SE: Standard error

### Associations between VEGF mRNA isoforms and levels of inflammatory molecules extracted from PBMCs

The Randox cytokine array detects four most common VEGF isoforms, however it does not quantify them individually but displays a sum of all VEGF detected in the sample. It has been previously confirmed that various isoforms portray different roles in pathophysiological processes; this is why their quantification and association to other inflammatory molecules are of particular interest. As a result, we investigated associations between 11 inflammatory molecules derived from PBMCs extracts and the expression of specific *VEGF* mRNA isoforms derived and quantified from PBMCs in a subset of individuals from the SFS cohort (Table 4).

**Table 4:** Characteristics of the subsample of the population used for assessment of associations between inflammatory molecules levels from PBMCs extracts and *VEGF* mRNA isoforms (n = 110).

	Mean	SD
**Age (years)**	47.35	10.78
**Gender (%) male**	48.18	-
**Interleukin 1 alpha (pg/mL)**	0.81	2.75
**Interleukin 1 beta (pg/mL)**	4.88	17.42
**Interleukin 2 (pg/mL)**	0.90	1.3
**Interleukin 4 (pg/mL)**	7.75	1.55
**Interleukin 6 (pg/mL)**	0.614	1.22
**Interleukin 8 (pg/mL)**	67.65	135.91
**Interleukin 10 (pg/mL)**	0.65	0.24
**Interferon gamma (pg/mL)**	0.30	0.36
**Tumour Necrosis Factor alpha (pg/mL)**	3.85	16.55
**Monocyte Chemoattractant Protein 1 (pg/mL)**	9.58	5.59
**Epidermal Growth Factor (pg/mL)**	6.84	7.09
***VEGF 121***	47.96	19.95
***VEGF 145***	47.19	22.52
***VEGF 165***	250.52	111.77
***VEGF 189***	15.03	7.17

VEGF: Vascular endothelial growth factor, VEGF transcript copies number/25 ng cDNA

Studies have established that *VEGF*_*165*_ and *VEGF*_*121*_ are the most abundantly expressed VEGF isoforms [38, 39]. Our detection of mRNA isoforms of VEGF in PBMCs has confirmed these findings.

**Fig 3:**
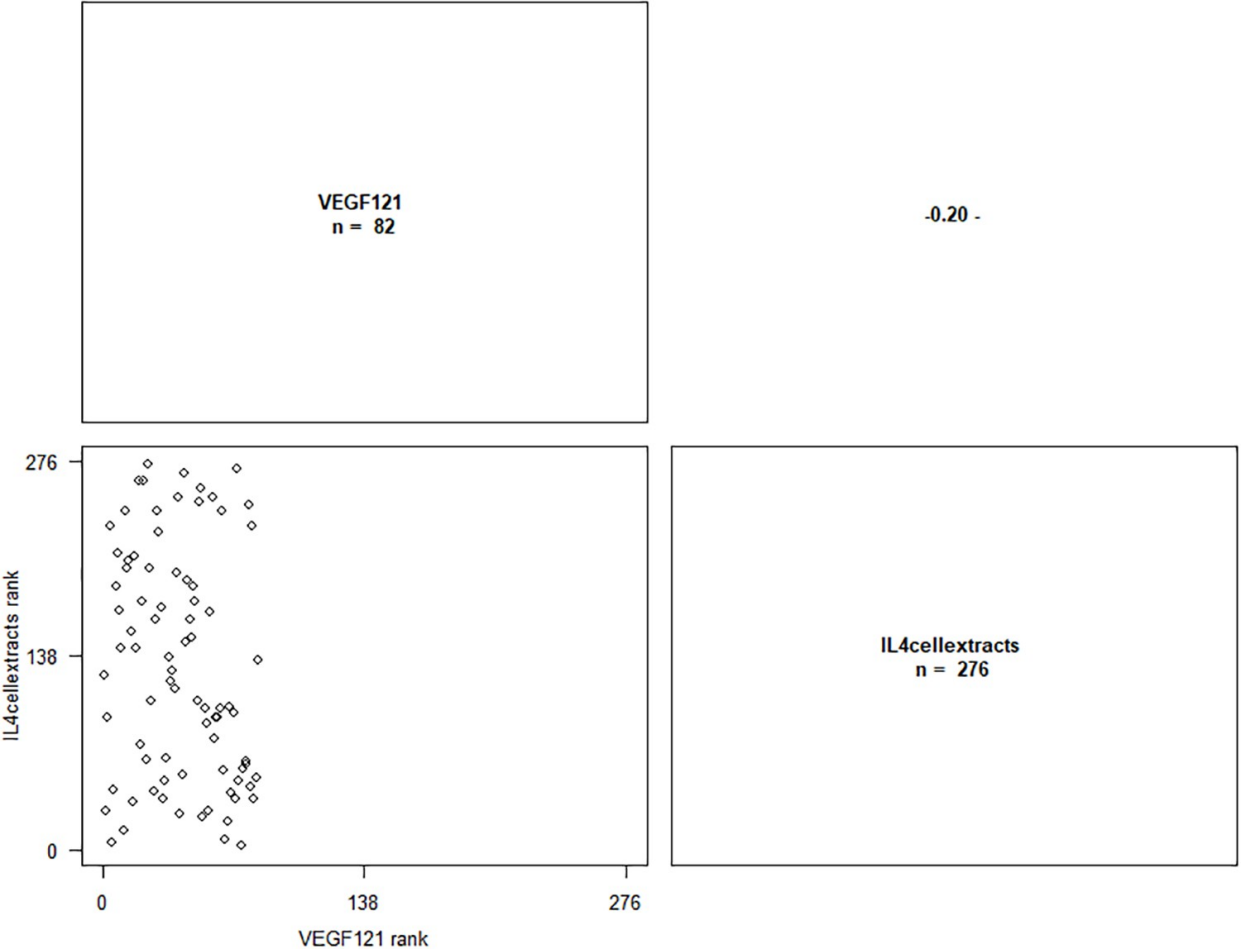
Correlation graph between *VEGF*_*121*_ isoform mRNA levels and IL-4 cellular extracts levels. ‘-’ p ≤ 0.1.

**Fig 4:**
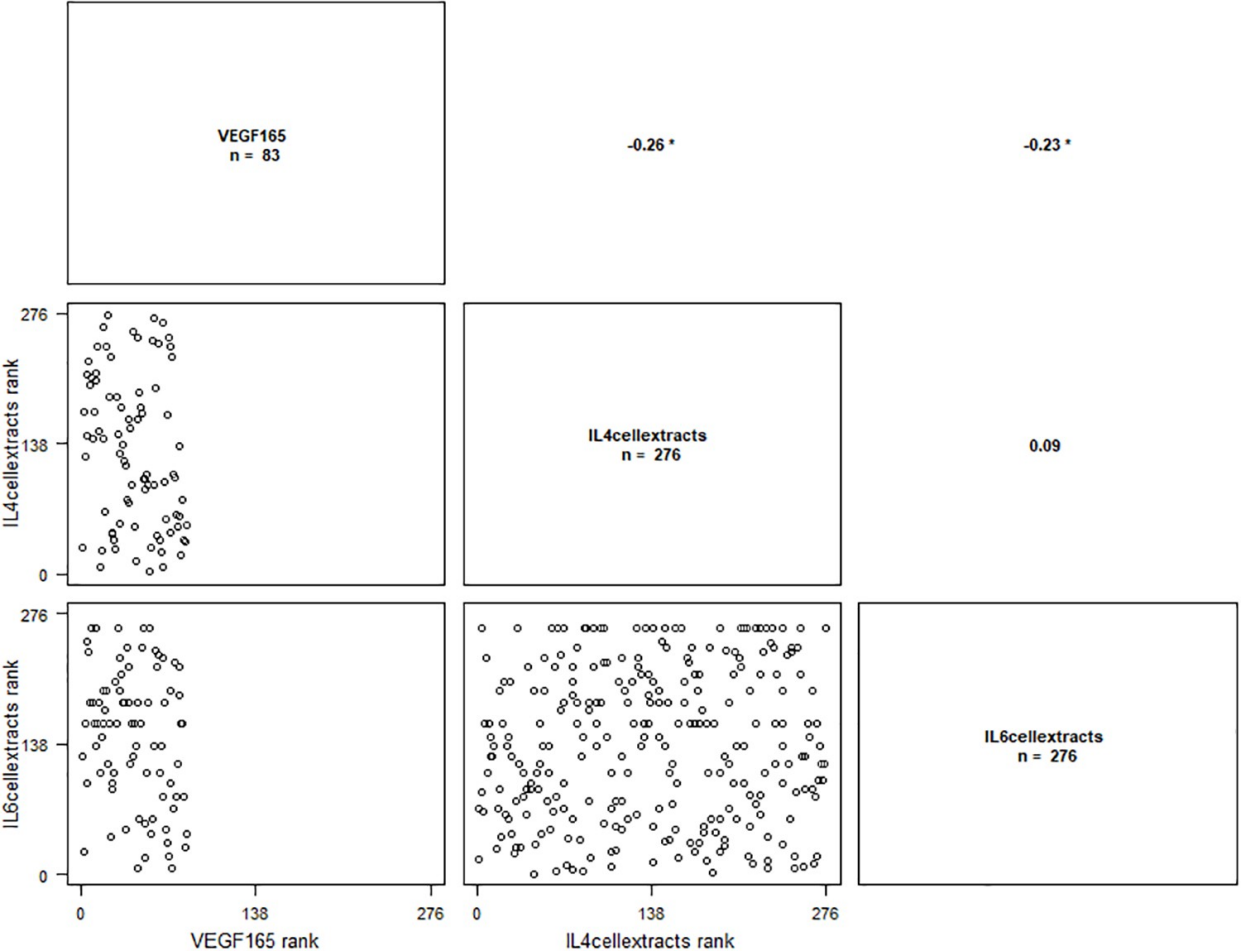
Correlation graph between *VEGF*_*165*_ isoform mRNA levels and IL-4 and IL-6 cellular extracts levels. ‘*’ p ≤ 0.05.

**Fig 5:**
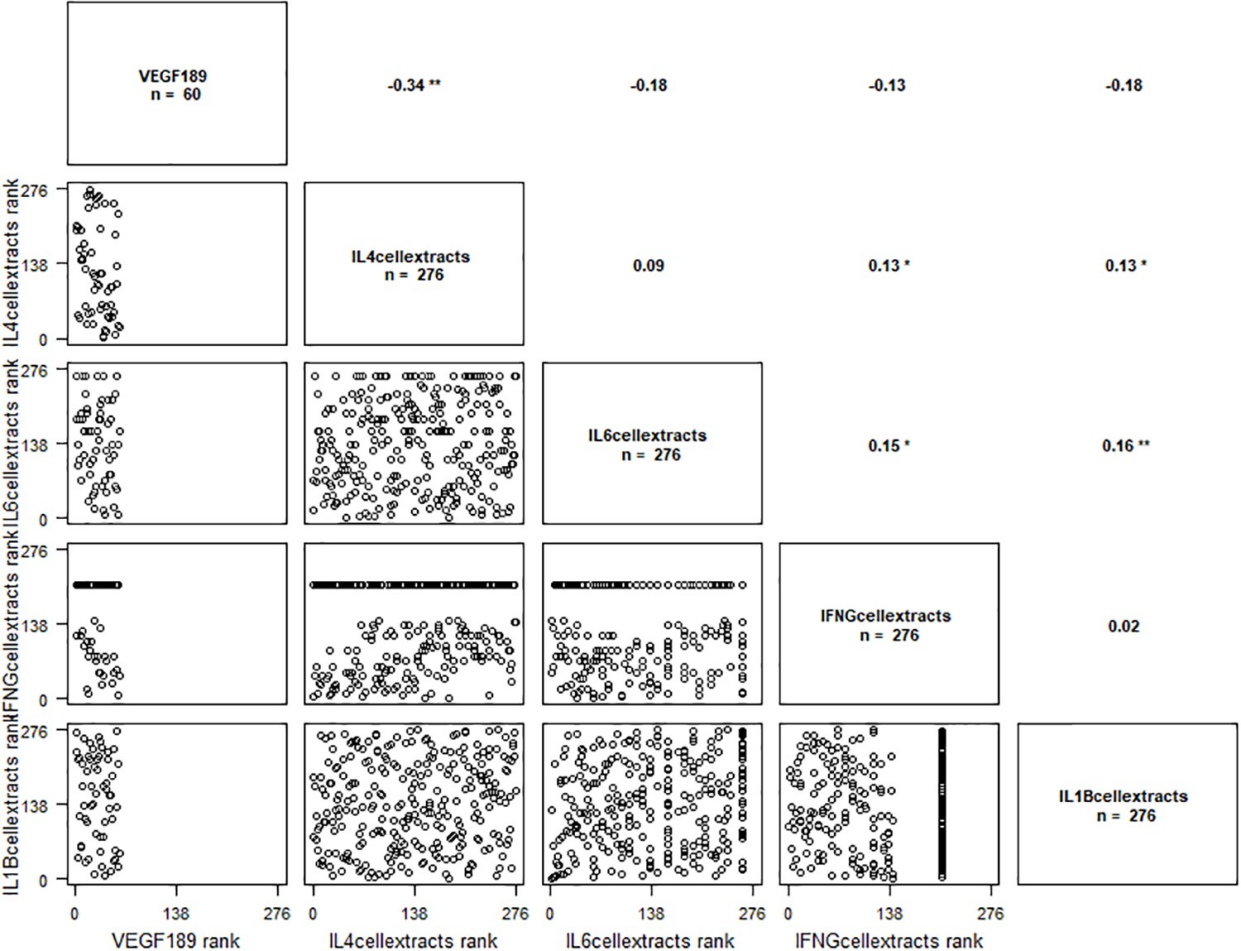
Correlation graph between *VEGF*_*189*_ isoform mRNA levels and IL-4, IL-6, INFγ et IL-1β cellular extracts levels. ‘**’ p ≤ 0.01, ‘*’p ≤ 0.05.

*VEGF*_*121*_ isoform was correlated with IL-4 (Fig 3), *VEGF*_*16*5_ isoform was correlated with IL4 and IL6 (Fig 4) and *VEGF*_*189*_ isoform was correlated with IFN-γ, IL-1β, IL-4 and IL-6 (Fig 5). Results are presented in Table 5, the significant P-values are presented in bold.

**Table 5:** Correlation analysis between VEGF isoforms and inflammation molecules.

	*VEGF*_*121*_		*VEGF*_*145*_		*VEGF*_*165*_		*VEGF*_*189*_	
	Correlation coefficient	P-value	Correlation coefficient	P-value	Correlation coefficient	P-value	Correlation coefficient	P-value
**EGF**	-0.1626	0.2748	-0.0607	0.6855	-0.1426	0.3388	-0.2128	0.1509
**IFN-γ**	-0.2412	0.1025	0.0034	0.9821	-0.2872	0.0503	-0.3415	**0.0188**
**IL-1α**	0.1276	0.3928	-0.236	0.1102	0.1256	0.4001	-0.0042	0.9778
**IL-1β**	-0.171	0.2504	-0.1735	0.2435	-0.1907	0.1992	-0.3088	**0.0347**
**IL-2**	-0.1759	0.237	0.129	0.3874	-0.1727	0.2457	-0.1155	0.4393
**IL-4**	-0.4345	**0.0023**	-0.1329	0.3731	-0.3643	**0.0118**	-0.3302	**0.0234**
**IL-6**	-0.2215	0.1346	-0.1797	0.2269	-0.3769	**0.009**	-0.3368	**0.0206**
**IL-8**	-0.0797	0.5942	0.0147	0.9216	-0.1354	0.3641	-0.1641	0.2702
**IL-10**	-0.0535	0.7208	-0.0787	0.5992	-0.0619	0.6794	-0.0676	0.6518
**MCP1**	-0.008	0.9577	-0.1209	0.4184	-0.0007	0.9963	-0.2326	0.1156
**TNF-α**	-0.0662	0.6584	0.0679	0.6503	-0.1271	0.3944	-0.2303	0.1194

**Fig 6:**
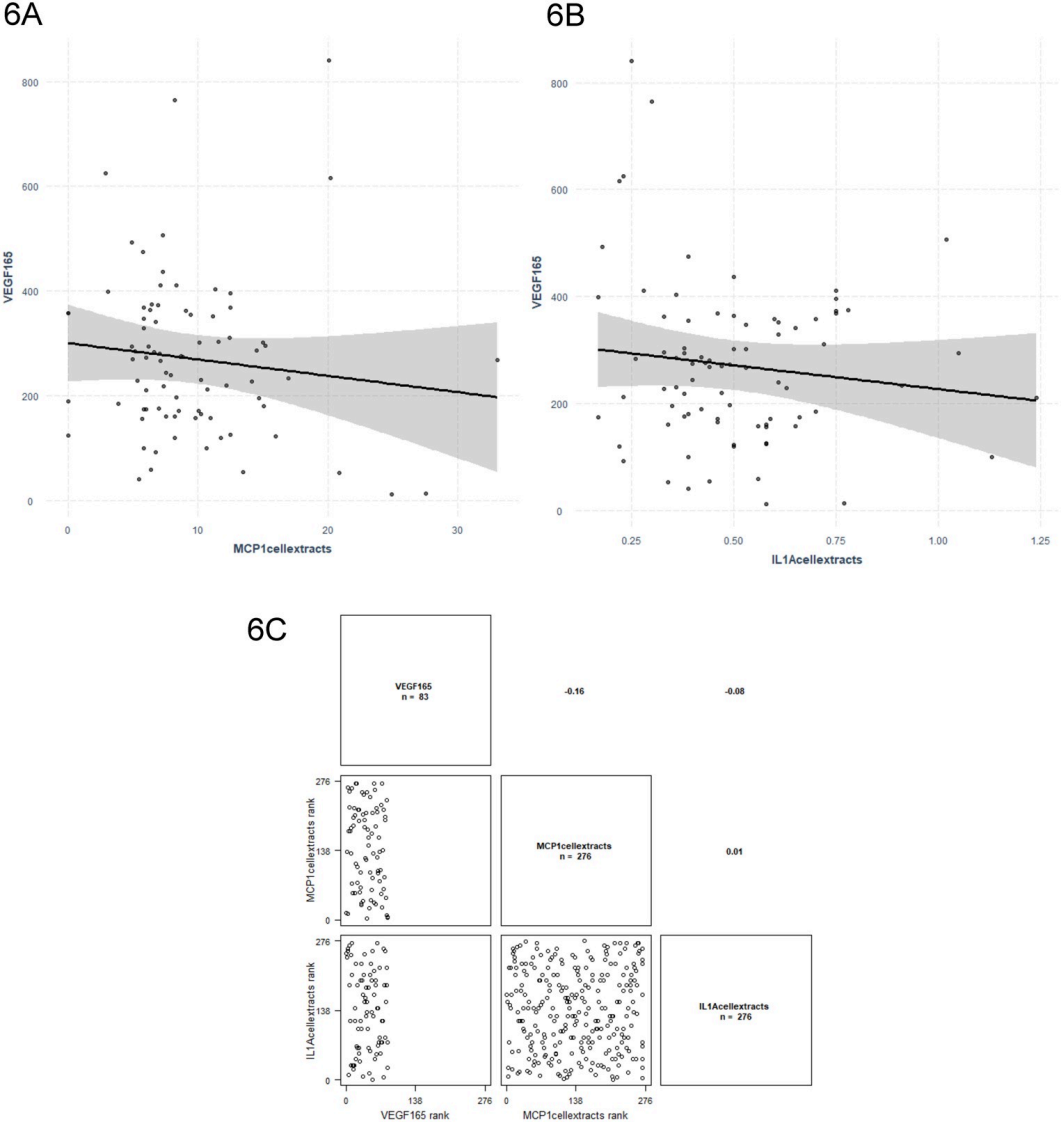
Regression graphs for *VEGF*_*165*_ isoform mRNA levels as dependent variable and MCP1 (6A) and IL-1α (6B) cellular extracts levels as independent variables. The related correlation graph is presented in (6C). ‘**’ p ≤ 0.01.

**Fig 7:**
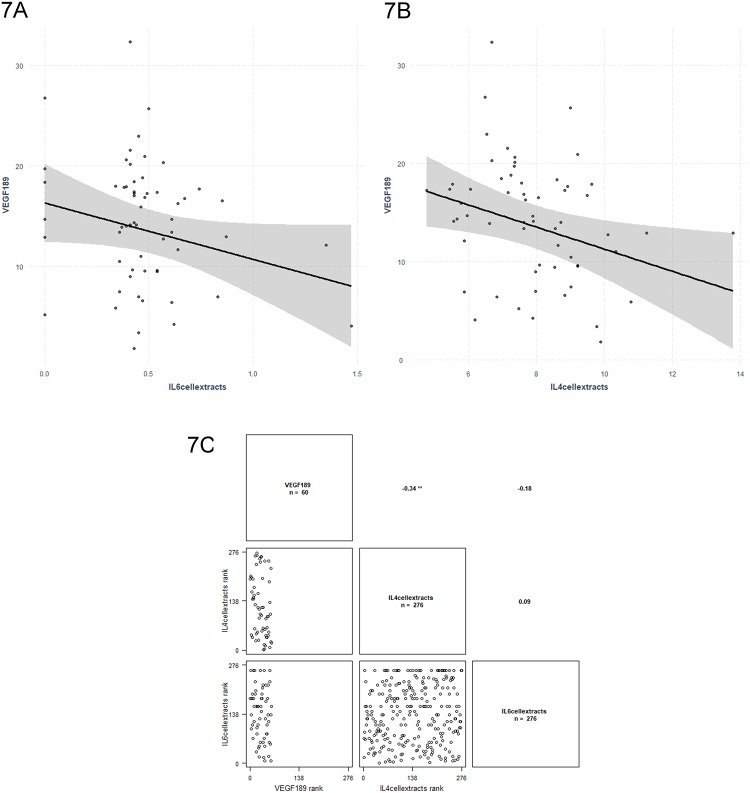
Regression graphs for *VEGF*_*189*_ isoform mRNA levels as dependent variable and IL-6 (7A) and IL-4 (7B) cellular extracts levels as independent variables. The related correlation graph is presented in (7C).

Two *VEGF* mRNA isoforms were significantly associated with four inflammatory molecules in linear regression analysis. *VEGF*_*165*_ was associated with MCP-1 (*P* = 0.002) and IL-1α (*P* = 0.008) (Fig 6), whereas *VEGF*_*189*_ was associated with IL-4 (*P* = 0.019) and IL-6 (*P* = 0.034) (Fig 7). Results are presented in Table 6.

**Table 6:** Significant associations between the expression of *VEGF* mRNA isoforms and inflammatory molecules (linear regression models adjusted for age and gender).

	*VEGF*_*165*_			*VEGF*_*189*_		
Inflammatory molecule	β	*SE*	*P*	β	*SE*	*P*
**Monocyte Chemoattractant Protein 1**	-0.319	0.006	0.002	-	-	-
**Interleukin 1 alpha**	-0.269	0.010	0.008	-	-	-
**Interleukin 4**	-	-	-	-0.290	0.017	0.019
**Interleukin 6**	-	-	-	-0.260	0.110	0.034

VEGF: *Vascular endothelial growth factor*

The STANISLAS cohort used in this study is composed of related individuals, therefore the analysis have been repeated using mixed models adjusted for family structure. The mixed model gave the same significant results as the linear regression model.

### Multiple alignment of VEGF isoforms

**Fig 8:**
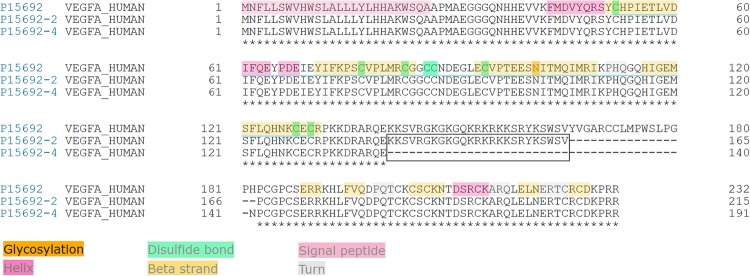
Alignment and elements of secondary structure of three VEGF isoforms (82% identity).

Analysis of primary and secondary structures of VEGF isoforms provides elements of understanding that may explain the commitment to different signaling pathways. VEGF_189_ and VEGF_165_ represent the same protein and differ only by 25 amino acids (Fig 8).

P15692 corresponds to *VEGF*_*206*_ considered as canonical sequence of VEGF. P15692-2 represents *VEGF*_*189*_ and P15692-4 represents *VEGF*_*165*_. Annotations are only depicted on the canonical sequence and stars indicate perfect alignment. The black frame shows the difference in amino acid composition (25) between *VEGF*_*189*_ and *VEGF*_*165*_.

**Fig 9:**
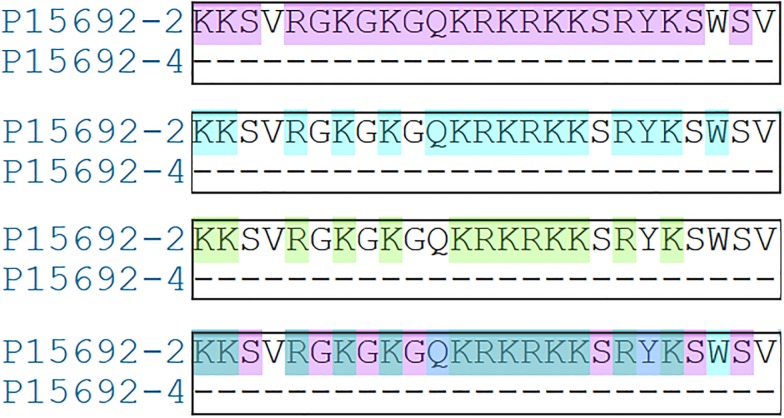
Focus on the 25 specific amino acids of *VEGF*_*189*_ versus *VEGF*_*165*_.

Thus, *VEGF*_*189*_ compared to *VEGF*_*165*_ owns additional residues, allowing to interact with molecules, such as IL-4 and IL-6. Without them, the signaling pathway seems to implicate others actors, such as MCP-1 and IL-1α. A detailed analysis shows that these surnumerary amino acids harbor specific properties (Fig 9).

P15692-2 corresponds to *VEGF*_*189*_ and P15692-4 to *VEGF*_*165*_. Analysis of amino acid properties revealed that *VEGF*_*189*_ owns 25 amino acids with three features. First, 22 are polar (top alignment highlighted in purple), second, 16 belong to the category of bulky amino acids (highlighted in blue), and third, 13 have a positive charge (highlighted in green). The last one is a superposition of the three.

These additional residues may modify polarity, steric hindering and the electrostatic potential of *VEGF*_*189*_ leading to change its counterparts and providing elements that could explain why these two close proteins are not associated with the same molecules.

## Discussion

In the present study, we explored the relationship between VEGF protein levels and inflammatory molecules derived from PBMCs extracts from healthy individuals from the SFS cohort. In addition, specific mRNA VEGF isoforms from PBMCs were assessed for association with inflammatory molecules. To the best of our knowledge, this is the first study to investigate levels of inflammatory molecules in PBMCs extracts and their relationship with VEGF protein levels and specific VEGF mRNA isoforms.

In summary, VEGF protein levels were correlated with EGF, IL1-β, IL-8, MCP1 and TNFα levels, while linear regression model adjusted for age and gender demonstrated that IL-4 (*P* = 0.013), MCP-1 (*P*≤0.0001) and EGF (*P*≤0.0001) levels were significant predictors of VEGF levels. The mRNA *VEGF*_*121*_ isoform was correlated with IL-4 levels, while the *VEGF*_*165*_ was correlated with IL-4 and IL-6 levels and the *VEGF*_*189*_ was correlated with IFN-γ, IL-1β, IL-4 and IL6 levels. The regression models adjusted for age and sex demonstrated that for the expression of the mRNA *VEGF*_*165*_ isoform the MCP-1 (*P* = 0.002) and IL-1α (*P* = 0.008) levels were significant predictors, while for the expression of the mRNA *VEGF*_*189*_ isoform, the IL-4 (*P* = 0.019) and IL-6 (*P* = 0.034) levels were significant predictors.

PBMCs are particularly interesting cells, worthy of investigation because of their essential role in the synthesis and release of inflammatory cytokines and growth factors. Therefore, it is biologically plausible to explore associations of inflammatory molecules with VEGF protein levels and specific *VEGF* mRNA isoforms (*i*.*e*. *VEGF*_*121*_, *VEGF*_*145*_, *VEGF*_*165*_ and *VEGF*_*189*_) derived from PBMCs. PBMCs are a complement of cells derived from whole blood, composed of lymphocytes, monocytes, and dendritic cells. In humans, the frequencies of these cell populations vary across individuals. Lymphocytes are typically in the range of 70% to 90% of total PBMCs, monocytes range from 10% to 30% of total PBMCs, while dendritic cells are uncommon, estimated at only 1% to 2% of total PBMCs [16].

PBMCs are commonly used in immunology to examine cytokine secretion under modified condition [40, 41], in vaccine development [42, 43] and can serve as tissue for gene expression studies [44]. Gene expression of *VEGF* isoforms has been systematically measured in PBMCs for different purposes; to compare the expression of different isoforms in particular disease [45, 46] or to understand the control of *VEGF* expression [47]. Besides *VEGF* expression profile, PBMCs have been used to detect RNAs for characterization of cardiovascular diseases [48, 49], diabetic nephropathy [50], rheumatic diseases [51], infectious diseases [52] and many other diseases. Protein expression of inflammatory cytokines from PBMCs has also been extensively studied. Increased expression of TNF-α and IL-6 along with increased secretion of leptin was found in obese cardiovascular patients [53]. Moreover, studies showed that protein expression or plasmatic levels of cytokines released by PBMCs can be indicators of pathologic state of organism [54]. Indeed, inflammation plays a central role in development of many chronic diseases [55] and PBMCs play an important role in inflammatory cytokine production.

Our investigation exploring the association between VEGF and inflammatory molecules followed a two-staged approach. Firstly, VEGF protein levels detected by microarray were analyzed. However, this method is not sensitive to specific VEGF isoforms; therefore, we used PBMCs also for mRNA isolation and quantification of *VEGF* expression isoforms. This enabled quantification of the expression of four isoforms in the second stage of the study. Two of the inflammatory molecules, IL-4 and MCP-1, were significantly associated to VEGF protein levels, and were also associated with the expression of *VEGF* specific isoforms 189 and 165, respectively.

Firstly, IL-4 was related with VEGF levels derived from PBMCs extracts *and VEGF*_*189*_ mRNA levels detected from PBMCs. IL-4 is a multi-functional cytokine with anti-inflammatory and anti-tumor activity, produced by lymphocytes, basophils and mast cells [56]. Its anti-inflammatory properties are due to the ability of controlling the production of pro-inflammatory mediators by inhibiting their induction [57]. IL-4 has been previously related to VEGF as an important factor in the recruitment of tumor-associated macrophages (TAMs), which are known to promote angiogenesis, tissue remodeling and immunosuppression. As it was demonstrated in the study of Linde et. al [23, 58], VEGF is responsible for recruitment of monocytes from the peripheral circulation, whereas IL-4 induces their differentiation into tumor-promoting M2-like macrophages. The association of VEGF and IL-4 was also detected in human coronary artery endothelial cells [59] and in smooth muscle cells [25]; both studies noticed induced release of VEGF while treated with IL-4. Faffe et al. [25] also reported the increasing of VEGF-mRNA expression, caused by IL-4, but observed no alteration of VEGF promoter activity. Additionally, IL-4 has been shown to increase VEGF levels in fibroblast-like synoviocytes when applied alone, but portrayed an anti-angiogenic effect in the presence of transforming growth factor (TGF)-β, by inhibiting the VEGF production, in the study of patients with rheumatoid arthritis [56]. Thus, these results demonstrate the importance of understanding the synergy of different molecules, which can elucidate important biological pathways and thus open new possibilities for treatment of complex diseases. Previous studies have not proposed a specific VEGF isoform related to IL-4. Of note, our study is the first to propose a link between the isoform 189 and IL-4, but an additional research is warranted to confirm this finding.

Secondly, MCP-1 was another molecule statistically associated with both VEGF protein levels and *VEGF* mRNA. MCP-1 is an angiogenic chemokine, involved in the regulation, migration and infiltration of monocytes and macrophages [60]. Previous research found that MCP-1 induced angiogenesis is mediated by VEGF [61]. More specifically, the researchers identified that MCP-1 up-regulated hypoxia-inducible factor 1 alpha gene expression in human aortic endothelial cells, which as a result induced *VEGF*_*165*_ expression in the aortic wall. The importance of MCP-1 in regulation of angiogenesis and immune system was discussed in the study of breast cancer tissue, where twelve inflammatory molecules were measured and evaluated for correlations [62]. This study confirmed that the expression of MCP-1 was correlated significantly with TAM accumulation in primary breast tumours, and with VEGF. Prognostic analysis revealed that high expression of MCP-1/VEGF was an independent indicator of early relapse of cancer. A study in vascular smooth muscle cells of rats [63] demonstrated that VEGF was responsible for the mitogenic activity of MCP-1, which leads to vessel wall remodeling. Moreover, severe hypoxia, previously known to induce VEGF release, potentiated the growth-promoting effect of MCP-1 [63]. This effect on vessel growth was studied also for therapeutic purposes. The therapy with dual delivery of VEGF and MCP-1 showed beneficial effects during transplantation of endothelial cells for therapeutic vascularisation [64], where VEGF improved survival of transplanted cells and MCP-1 induced mural cell recruitment. In the current study, MCP-1 was associated with VEGF protein levels and specific mRNA *VEGF*_*165*_ isoform, which confirmed the results of previous studies.

Thirdly, our study identified association of VEGF isoform 165 also with protein IL-6. Several studies demonstrated IL-6 induction of *VEGF* expression. For example, Choen et al. demonstrated that treatment of cell lines with IL-6 results in significant increase of *VEGF* mRNA [65]. Moreover, IL-6 was shown to promote cervical tumorigenesis by activating VEGF-mediated angiogenesis via a STAT3 pathway [66]. Similarly, it has been shown to induce VEGF expression and as a result increase angiogenesis in gastric carcinoma patients [26] and in malignant mesothelioma [67]. These results suggest that treatment with IL-6R antibody might constitute a potential target therapy for certain cancer types. We would, again, reiterate the importance of testing for specific *VEGF* isoforms, which would, in combination with different targeted proteins, such as IL-6, contribute to specific and efficient therapies.

An association was observed also between IL-1α and *VEGF*_*165*_ isoform. IL-1α is a prototypical proinflammatory cytokine, known also as hematopoietin 1, which is involved in various immune responses, inflammatory processes, and hematopoiesis [68]. It is produced by monocytes and macrophages and released in response to cell injury [69]. It has been previously shown that IL-1α stimulates VEGF secretion by PBMCs in a dose-depended manner, via induction of *VEGF* mRNA synthesis. Four VEGF isoforms were encoded with *de novo* synthetized mRNA, *VEGF*_*121*_, *VEGF*_*165*_, *VEGF*_*189*_, and *VEGF*_*206*_. Moreover, *VEGF*_*121*_ and *VEGF*_*165*_ gave the major signals both in unstimulated and IL-1α stimulated PBMCs, which is also in favour of our results [21]. Correlation of VEGF production with IL-1α and IL-6 in cell lines suggests that the expression of VEGF is regulated by IL-1α and IL-6 in pancreatic cancer [70] and in adenoma cells [71].

Finally, an association of VEGF protein levels and EGF has been observed in the current study. EGF plays an important role in the growth, proliferation and differentiation of numerous cell types, by binding with high affinity to the cell surface receptor [72]. Major role of both related growth factors in cancer development has been researched for various types of cancers and different mechanism of their common pathways have been proposed [73–75]. Combination of VEGF/EGF signaling pathways has been proposed for new therapeutic approaches of anticancer therapy [76–79] and is showing positive predictions for future development of new medications.

VEGF isoforms are expressed in different quantities, based on the cell type that is producing them. Most cells express *VEGF*_*121*_, *VEGF*_*165*_ (the most abundant) and *VEGF*_*189*_. Quantification of VEGF isoforms in our study confirmed the reports from literature, as the most expressed isoform from the PBMCs population was *VEGF*_*165*_, followed by *VEGF*_*121*_. *VEGF*_*145*_ was also quantified in our research, although it is more commonly found in cells of placental origin [80, 81]. The notion that the identification of VEGF isoforms is important in fundamental biological and pharmacological studies has arisen the last few years. Specific VEGF isoforms were studied in different types of cancer [82], cardiovascular disease [83], kidney disease [84], autoimmune disease [85] and many others. Pro-angiogenic VEGFxxx and anti-angiogenic VEGFxxxb splicing variants are particularly interesting; it has been demonstrated that their ratio varies based on different pathology, therefore, determination of pro and anti-angiogenic isoforms could serve as diagnostic tools for such diseases [86–88]. Moreover, the quantification of pro and anti-angiogenic isoforms was shown to have a predictive value in response to a treatment with bevacizumab [89]. However, there is no available method that could separate pro and anti-angiogenic isoforms routinely, therefore, most of the studies quantify only pro-angiogenic isoforms. Likewise, in our research, only pro-angiogenic isoforms were detected and associated with specific protein levels. Still, we are aware of the importance of separation and quantification of all VEGF splice variants. We believe that in the future studies, anti-angiogenic isoforms should be routinely quantified and distinguished from pro-angiogenic isoforms for attempting diagnosis of different chronic diseases and prediction of response of anti-VEGF agents (pharmacogenomics).

With such progress, the discovery of anticancer treatments, which will specifically target VEGF isoforms and would decrease the risk of adverse effects of current anti-VEGF therapies will be only a matter of time. VEGF isoforms have already shown a good potential for significant and independent roles in the prediction of different types of cancers [90, 91] and differentiation of isoforms for therapeutic purposes is now being extensively studied [92, 93]. On the other hand, in the complex system of biological pathways, there is usually more than one factor impacting on a pathological development. Therefore, knowing the roles of various proteins related to VEGF is of special importance. It has been shown in previous studies that many cytokines and growth factors upregulate *VEGF* mRNA or induce VEGF release [31]. In support of this, the current investigation also identifies several significant associations.

*VEGF*_*189*_ and *VEGF*_*165*_ are two isoforms of VEGF protein sharing more than 88% sequence identity. Indeed, analysis of their multiple alignment shows that they differ by only 25 amino acids. These additional amino acids modifying polarity, steric hindering and basic properties confer a subtle difference, sufficient to significantly modify their features and involve them in different signaling pathways. Thus, according to our results, thanks to supplemental residues *VEGF*_*189*_ could favor molecules interacting with IL-4 and IL-6 while *VEGF*_*165*_ seems to be associated rather with MCP-1 and IL-1α.

One main limitation of our study is that we were unable to confirm the obtained results for VEGFmRNA/protein levels as the availability of biological materials was unfortunately limiting.

## Conclusion

This investigation has revealed significant associations between VEGF levels and its specific mRNA isoforms with inflammatory molecules derived from PBMCs cellular extracts, specifically cytokines and growth factors IL-1α, IL4, IL-6, EGF, and MCP1 in a population of healthy individuals from the SFS. This confirmed that cytokines and growth factors play an important role in VEGF regulation also at lymphocytes cellular extract level. Importantly, specific isoforms interact with different inflammatory molecules. Therefore, they should be identified in future investigations of molecular and biological pathways involving VEGF, which would contribute to an in-depth and specific understanding of the process and would ultimately offer novel targeted therapies for VEGF-related pathophysiology.

## Supporting information

S1 Table Primer sequences for quantification of VEGF transcripts. (DOCX)

S1 Data Supporting information—Data set. (XLSX)
