# Correction: Peripheral blood mononuclear cells extracts VEGF protein levels and VEGF mRNA: Associations with inflammatory molecules in a healthy population

**DOI:** 10.1371/journal.pone.0224591

**Published:** 2019-10-24

**Authors:** Vesna Gorenjak, Dwaine R. Vance, Alexandros M. Petrelis, Maria G. Stathopoulou, Sébastien Dadé, Said El Shamieh, Helena Murray, Christine Masson, John Lamont, Peter Fitzgerald, Sophie Visvikis-Siest

The sixth author’s initials appear incorrectly in the citation. The correct citation is: Gorenjak V, Vance DR, Petrelis AM, Stathopoulou MG, Dadé S, El Shamieh S, et al. (2019) Peripheral blood mononuclear cells extracts VEGF protein levels and VEGF mRNA: Associations with inflammatory molecules in a healthy population. PLoS ONE 14(8): e0220902. https://doi.org/10.1371/journal.pone.0220902
