# Supplementary material for: Peripheral blood mononuclear cells extracts VEGF protein levels and VEGF mRNA: Associations with inflammatory molecules in a healthy population
Source: PLoS One. 2019 Aug 16;14(8):e0220902. doi: 10.1371/journal.pone.0220902 (PMC6697334; doi:10.1371/journal.pone.0220902)
Supplement: S1 Table — (DOCX) [file pone.0220902.s001.docx]

**Table S1: Primer sequences for quantification of *VEGF* transcripts.**

| *VEGF* Isoform | Primer | Sequence |
| --- | --- | --- |
| *VEGF* | Sense | 5’-GAGCTTCCTACAGCACAACAAA-3’ |
|  |  |  |
| *VEGF121* | Anti-sense | 5′-CTCGGCTTGTCACATTTTTC-3′ |
|  | Probe | 5′-TGCAGACCAAAGAAAGATAGCAAGACA-3′ |
|  |  |  |
| *VEGF145* | Anti-sense | 5′-CTTGTCACATACGCTCCAGGAC-3′ |
|  | Probe | 5′-AAACGAAAGCGCAAGAAATCCCGGTA-3′ |
|  |  |  |
| *VEGF165* | Anti-sense | 5′-GCTTTCTCCGCTCTGAGCA-3′ |
|  | Probe | 5′-AGCAAGACAAGAAAATCCCTGTGGGCC-3′ |
|  |  |  |
| *VEGF189* | Anti-sense | 5′-CCACAGGGAACGCTCCAGGAC-3′ |
|  | Probe | 5′-AGCAAGACAAGAAAAAAAATCAGTTCGAGGAAA-3′ |
